# Nanoparticles for Biomedical Use Derived from Natural Biomolecules: Tannic Acid and Arginine

**DOI:** 10.3390/biomedicines13010209

**Published:** 2025-01-16

**Authors:** Mehtap Sahiner, Selin S. Suner, Nurettin Sahiner

**Affiliations:** 1 Department of Bioengineering, Faculty of Engineering, Canakkale Onsekiz Mart University, Terzioglu Campus, Canakkale 17199, Turkey; sahinerm78@gmail.com; 2 Department of Chemistry, Faculty of Sciences, Canakkale Onsekiz Mart University, Terzioglu Campus, Canakkale 17100, Turkey; sagbasselin@gmail.com; 3 Department of Bioengineering, U.A. Whitaker College of Engineering, Florida Gulf Coast University, Fort Myers, FL 33965, USA

**Keywords:** tannic acid, arginine, nanoparticles, phenolic- and amino acid-based particles, antioxidant, antimicrobial, nanogel/microgel

## Abstract

**Background/Objectives**: Tannic acid (TA) is a well-known natural phenolic acid composed of ten gallic acids linked to each other with ester bonding possessing excellent antioxidant properties in addition to antimicrobial and anticancer characteristics. Arginine (ARG) is a positively charged amino acid at physiological pH because of nitrogen-rich side chain. **Method**: Here, poly(tannic acid-co-arginine) (p(TA-co-ARG)) particles at three mole ratios, TA:ARG = 1:1, 1:2, and 1:3, were prepared via a Mannich condensation reaction between TA and ARG by utilizing formaldehyde as a linking agent. **Results**: The p(TA-co-ARG) particles in 300–1000 nm size range with smooth surfaces visualized via SEM analysis were attained. Abundant numbers of functional groups, -OH, -NH_2_, and -COOH stemming from TA and ARG constituent confirmed by FT-IR analysis. The isoelectric point (IEP) of the particles increased from pH 4.98 to pH 7.30 by increasing the ARG ratios in p(TA-co-ARG) particles. The antioxidant capacity of p(TA-co-ARG) particles via gallic acid (GA) and rosmarinic acid (RA) equivalents tests revealed that particles possess concentration-dependent antioxidant potency and increased by TA content. The α-glucosidase inhibition of p(TA-co-ARG) particles (2 mg/mL) 1:1 and 1:2 mole ratios revealed significant enzyme inhibition ability, e.g., 91.3 ± 3.1% and 77.6 ± 12.0%. Interestingly, p(TA-co-ARG) (1:3 ratio) possessed significant antibacterial effectiveness against *Escherichia coli* (ATCC 8739) and *Staphylococcus aureus* (ATCC 6538) bacteria. Furthermore, all p(TA-co-ARG) particles at 1000 mg/mL concentration showed >80% toxicity on L929 fibroblast cells and increased as ARG content of p(TA-co-ARG) particles is increased. **Conclusions**: p(TA-co-ARG) showed significant potential as natural biomaterials for biomedical use.

## 1. Introduction

Nanogels, which are nano-sized hydrogels up to a few 100 nm, have a high capacity for hydration and can expand or contract more rapidly to a variety of stimuli depending on the functional groups they possess. These functional groups enable them to link or encapsulate hydrophobic or hydrophilic medications to prevent them from degrading in the body or during storage [1]. The advantages of hydrogels and nanoparticles are combined in nanogels or microgels, which are generally prepared from synthetic or natural polymers or monomers or molecules. Nanogels are also crosslinked structures (at the nanoscale level) that can hold large volumes of water or other fluids without dissolving, making them more biocompatible [2]. The biocompatibility of nanogels mostly depends on the starting materials used in their synthesis, and it is reasonable to choose natural material with biocompatible nature to prepare nanogels for use in biomedical applications [3]. Many natural polymers such as chitosan, alginate, hyaluronic acid, and different polysaccharides and natural gums such as inulin, guar gum, agar gum, and so on are widely used in microgel/nanogel synthesis for a variety of biomedical applications [2,3,4].

There are some reports about nanoparticle preparation through the dissolution of different polyphenol structures, such as epigallocatechin in water; the formation of functional groups with formaldehyde; and a subsequent reaction with amino acids like lysine and glycine [4]. The linear polyphenol oligomer from, e.g., tea polyphenol derivatives, is also used in the production of a three-component process through the Mannich reaction self-assembly into polyphenol nanoparticles [5]. Recently, our group reported the use of a natural phenolic cell-staining dye molecule, hematoxylin, that is integrated with L-lysine (l) via a Mannich condensation reaction to create nanonetworks that self-assembled into nanoparticles with immense biomedical application potential [6].

The amino acid L-arginine (ARG) is synthesized in the kidney via the urea cycle. Cells that do not synthesize ARG alone can use cationic amino acid transporters. ARG serves as a substrate for nitric oxide synthase (NOS), which exists in three isoforms: inducible NOS, neuronal NOS, and endothelial NOS [7]. An essential amino acid, ARG is a precursor to the synthesis of proteins, urea, ornithine, citrulline, nitric oxide (NO), creatinine, agmatine, glutamate, proline, putrescine, spermidine, and spermine [8]. It also supports the metabolism of proline, glutamate, and polyamines at the level of the entire organism or, in the case of mammals, at the level of individual cells. The availability and metabolism of ARG could restore physiological equilibrium and regulate immune response and inflammation during infections [9]. Also, dimethylarginine and homoarginine, two endogenous arginine derivatives, are linked to chronic illnesses often seen in the elderly, including depression, dementia, and cardiovascular disease [10]. Methylation of arginine affects gene transcription and is crucial for regulating the mTOR pathway, which is important for cell growth [11]. Due to limited kidney synthesis, healthy adults mostly obtain arginine through diet. However, newborns, young children, and adults suffering from serious intestinal and kidney infections cannot create enough of this production. Although arginine supplements can improve a patient’s immune response after surgery, they may raise the risk of death for people who have had an acute myocardial infarction [12]. It is obvious that ARG has many biological functions, and its use as biomaterials would afford great advantages.

Tannic acid (TA) belongs to the group of phenolic acids and its subgroup is hydroxybenzoic acid [13]. TA is a natural compound that is abundant and inexpensive in acorns. It hydrolyzes into acidic residues and forms 10 molecules of gallic acid, which is known for its antioxidant properties [14,15]. TA is frequently being studied in biomedical materials due to its antimicrobial, antioxidant, and anticancer properties [16]. TA’s use as a coagulant in wound dressings due to its ability to clot blood is also another factor in its widespread biological applications [17]. Moreover, TA can be used to treat severe burns and gastrointestinal issues like diarrhea and hemorrhoids and to prevent neurodegenerative diseases [18].

Here, we use TA with ARG to prepare nanoparticles via self-assembling networks by combining them at various mole ratios to prepare p(TA-co-ARG) nanoparticles. The well-known Mannich condensation reaction was utilized using formaldehyde as a linking agent in the preparation of p(TA-co-ARG) nanonetworks. Size and structures of p(TA-co-ARG) nanogels were characterized using dynamic light scattering (DLS), Fourier transform infrared radiation (FT-IR) spectroscopy, thermogravimetric analysis (TGA), and SEM analysis. Moreover, the antioxidant properties using GA and RA antioxidant equivalent capability, cell cytotoxicity via an MTT assay against fibroblast cells, and α-glucosidase inhibition were tested. Finally, the antibacterial properties of p(TA-co-ARG) nanoparticles against Gram-negative *Escherichia coli* (ATCC 8739) and Gram-positive *Staphylococcus aureus* (ATCC 6538) bacteria were tested.

## 2. Materials and Methods

### 2.1. Materials

Tannic acid (TA, 97%, Sigma-Aldrich, St. Louis, MO, USA), L-arginine (ARG, 98%, Sigma-Aldrich), formaldehyde (FA, 37% aqueous solution, Sigma-Aldrich), and absolute ethyl alcohol (99%, Carlo-Erba GmbH, Emmendingen, Germany) were used as received in the preparation of p(TA-co-ARG) nanoparticles. Folin and Ciocalteu’s phenol reagent (FC, Sigma-Aldrich, St. Louis, MO, USA), sodium nitrite (Merck, extra pure, Rahway, NJ, USA), aluminum chloride anhydrous powder sublimed from synthesis (Merck, Darmstadt, Germany), gallic acid (GA, 97.5–102.5%, Aldrich, St. Louis, MO, USA), rosmarinic acid (RA, 96%, Aldrich, St. Louis, MO, USA), Fe(II) sulfate heptahydrate (Merck, 99.5%), and 5,6-Diphenyl-3-(2pyridyl)-1,2,4-triazine-4,4-disulfonic acid disodium salt hydrate (Alfa Aesar, Ward Hill, MA, USA) were employed in antioxidant assays. The α-glucosidase from *Saccharomyces cerevisiae* (100 unit/mg, Sigma-Aldrich, St. Louis, MO, USA) and 4-nitrophenyl-α-D-glucopyranose (99%, Acros Organics, Geel, Belgium) were used as the enzyme and substrate.

*Escherichia coli* (*E. coli*, ATCC 8739) as Gram-negative bacteria and *Staphylococcus aureus* (*S. aureus*, ATCC 6538) as Gram-positive bacteria (KWIK-STIK, Microbiologics, France) were used in the determination of the antibacterial potency of p(TA-co-ARG) particles. As a bacterial growing medium, nutrient agar (NA, Condolab, Madrid, Spain) and nutrient broth (NB, Merck, Darmstadt, Germany) were used as received. Fibroblast cells (L929, mouse C3/An connective tissue) were purchased from a national supplier (SAP Institute, Ankara, Turkey). In the cell culture, Dulbecco’s modified Eagle’s medium (DMEM, containing 4.5 g/L glucose, 3.7 g/L sodium pyruvate, and 0.5 g/mL L-glutamine), fetal bovine serum (FBS), antibiotic solution (100 IU/mL penicillin/100 μg/mL streptomycin), and trypsin/EDTA (0.25%Trypsin/0.02% EDTA) were obtained from PanBiontech (GmbH, Aidenbach, Germany). Trypan blue solution (0.5%, Biological Industries, Haifa, Isreal), 3-(4,5-dimethylthiazol-2-yl)-2,5-diphenyltetrazolium bromide (MTT agent, neoFroxx GmbH, Hesse, Germany), and dimethyl sulfoxide (DMSO, 99.9 %, Carlo-Erba GmbH, Emmendingen, Germany) were purchased and used as received. Aqueous solutions were prepared with DI water (Millipore-Direct Q UV3 at 18.2 M.Ω.cm).

### 2.2. Preparation of p(TA-co-ARG) Nanoparticles

A stock solution of ethanol:water (5:25) at a 1 L volume was prepared. At 550 rpm, 30 mL of stock solution was used to dissolve 0.1 g of TA. The reaction then proceeded for 30 min at 500 rpm after 25 mmol FA (37%) was added. After that, one mole, two mol, or three mol of ARG was added to the TA solution, separately dissolved into 3 mL of stock solution, and mixed the then left to react for 2.5 h. Then, the obtained particles were precipitated at 11,000 rpm for 15 min by centrifugation. After washing 3 times with DI water, a final wash with acetone was performed, and the particles were dried with a drying gun and stored in a closed container for further use.

### 2.3. Characterization of p(TA-co-ARG) Nanoparticles

The morphological structure of p(TA-co-ARG) particles was visualized by a scanning electron microscope (SEM, SU70, Hitachi, Japan). Before imaging, the particles were covered with a few μm of Pd/Au for 20 s, and the images were taken at a 5 kV operating voltage.

The size distribution of the p(TA-co-ARG) particles was measured using dynamic light scattering (DLS, Brookhaven Instrument Nanobrook Omni, Brookhaven, NY, USA) with a 35 mW solid-state laser detector at a wavelength of 658 nm.

A Fourier transform infrared radiation (FT-IR, Nicolet iS10, Thermo, Waltham, MA, USA) analysis of the p(TA-co-ARG) particles was carried out in the spectral range of 4000–650 cm^−1^ at a 4 cm^−1^ resolution using the ATR technique.

Zeta potential measurement at different pH conditions and isoelectronic points of the (TA-co-ARG) particles was determined by using a zeta potential analyzer (Brookhaven Instrument, BI-ZTU, Brookhaven, NY, USA). For the analysis, a 1 mg/mL p(TA-co-ARG) particle suspension was prepared in 1 mM KNO_3_ aqueous solution.

A thermogravimetric analyzer (TGA, SII TG/DTA 6300, Seiko, Tokyo, Japan) was used to investigate the thermal degradation properties of p(TA-co-ARG) particles in the temperature range of 100–700 °C at a 10 °C/min heating rate under N_2_ with a 2 mL/min flow rate.

### 2.4. Antioxidant and Enzyme Inhibition Effect of p(TA-co-ARG) Nanoparticles

In DI water, 2000 μg/mL p(TA-co-ARG) particles were suspended to be used in the total phenol test. Diluting the suspension solution multiple times from 1000 to 62.5 µg/mL was performed. An amount of 125 µL of FC solution and 20 µL of sample solution were put into a 96-well plate. Then, 100 µL of an aqueous Na_2_CO_3_ solution with a concentration of 0–7 M was added, and the mixture was then allowed to sit in the dark for two hours. The solutions’ absorbance value was then measured with a microplate reader (Thermo Scientific, Multiskan SKY, Waltham, MA, USA) at 760 nm. The antioxidant test result was reported as the gallic acid (GA) equivalent, as GA is considered as the standard reference material.

The total flavonoid (TFC) content of p(TA-co-ARG) particles was ascertained by testing their antioxidant characteristics. For this purpose, 50 µL of the suspended solution in the concentration range of p(TA-co-ARG) particles of 2000 µg/mL was added to 96 wells, followed by the addition of 25 µL of a 3% NaNO_2_ solution. Then, 6% AlCl_3_ solution was added to these wells. Finally, 100 μL of a 1 M NaOH solution was added to the wells. At 405 nm, the absorbance values of the solutions were compared with a previously established calibration of TPC values of rosmarinic acid (RA), and the results were expressed in µg/mL RA equivalents of total phenol.

The chelating activity of Fe(II) was studied according to the literature [19]. p(TA-co-ARG) particles were prepared at a concentration of 250 μg/mL in DI water. A sample solution of 140 μL was added to a 96-well plate, and 20 μL of 1 mM Fe(II) aqueous solution was added to each well. The plate was measured at 562 nm with a microplate reader (Thermo Multiskan Go, ThermoFisher, Waltham, MA, USA). After the measurement, 40 μL of a 2.5 mM ferrozine solution in deionized water was added to each well. After 5 min, the plate was measured again at 562 nm.

Enzyme inhibition studies were conducted using 4-nitrophenyl-α-D-glucopyranose as a substrate and the enzyme α-glucosidase as a model enzyme. The experiment was carried out in accordance with the literature [14]. In summary, 96 wells were filled with sample solutions containing 2000 mg/mL and 70 µL of 0.03 unit enzyme/mL. At 405 nm, the initial reading was taken. An amount of 70 µL of substrate solution was added after five minutes. Twenty minutes later, the last reading was obtained, the required computations were performed, and the % inhibition value was determined using the following formula.

Inhibition% = (1 − (A_sample_/A_control_)) × 100

When A_control_ is measured without added p(TA-co-ARG) particles and A_sample_ is measured with the added monomer or eluate.

### 2.5. Antibacterial Activity of p(TA-co-ARG) Nanoparticles

The antibacterial capability of the 1:1, 1:2, and 1:3 mole ratio of p(TA-co-ARG) particles was determined by microtiter broth dilution and disc diffusion tests against Gram-negative *Escherichia coli* (ATCC 8739) and Gram-positive *Staphylococcus aureus* (ATCC 6538).

In the microtiter broth dilution assay, 100 μL of nutrient broth (NB) was placed in each well of 96-well plates. Separately, a 50 mg/mL concentration of p(TA-co-ARG) particles was suspended in DI water, and 100 μL of this suspension was added to the first well. After that, this 25 mg/mL concentration of the first well was serially diluted to 0.2 mg/mL concentration by using NB solution. Then, 1.5 × 10^8^ CFU/mL, 5 μL of bacterial stock suspension was added to each well. As a negative control, 5 μL of bacteria suspension was added into 100 μL of nutrient broth (NB) solution in the absence of the sample. The plate was incubated at 37 °C for 18–14 h. At the end of the incubation time, the optical density (OD) was recorded at 590 nm by a microplate reader (Multiskan™ FC, Microplate Photometer, Thermo Fisher Scientific, Waltham, MA, USA) to assess the bacterial inhibition effects of the particles by comparing with the control. Minimum inhibition concentration (MIC) was determined by the lowest concentration of p(TA-co-ARG) particle suspension, which was observed to be transparent wells. After that, all transparent wells were inoculated on nutrient agar (NA) and incubated at 37 °C for 18–14 h. Then, the minimum bactericidal concentration (MBC) was evaluated by the lowest concentration of p(TA-co-ARG) particle suspension, which showed no bacterial growth.

In the disc diffusion assay, 1.5 × 10^8^ CFU/mL, 100 μL of bacterial stock suspension was inoculated on NA. Then, a 9 mm sterile square-shaped disc that consisted of 20 μL of 50 mg/mL p(TA-co-ARG) particle suspension was placed on NA. The agar plate was incubated at 37 °C for 18–14 h for bacteria growth, and the clear zone mm around the discs was measured to find the inhibition zone of the (TA-co-ARG) particles.

### 2.6. Cytotoxicity of p(TA-co-ARG) Nanoparticles

Toxicity on L929 fibroblasts was investigated for p(TA-co-ARG) particles using an MTT analysis. In the cell culture, 10% and 1% antibiotic-containing DMEM medium were used as a growth medium, and the cells were incubated at 5% CO_2_/95% air atmosphere at 37 °C. For the cytotoxicity analysis, nearly 100 μL of 1 × 10^4^ cell suspension in the medium was added into a 96 well-plate for each well and incubated for 24 h. After adhesion of the cell, 100 μL of the sterile p(TA-co-ARG) particle suspension in the medium at the 50–1000 μg/mL concentration range was added to the cells. The plate was incubated at the same condition for 24 h more. After that, the cells were gently washed with phosphate-buffered solution (PBS) and interacted with 100 μL of 0.5 mg/mL MTT reagent for 4 h in the dark. Then, 200 μL of DMSO was used to dissolve the formazan crystals into each well, and the absorbance of the plate was determined at a 570 nm wavelength by using a plate reader (Multiskan™ FC, Microplate Photometer, Thermo Fisher Scientific, Waltham, MA, USA). This analysis was repeated three times, and the results are given with standard deviations. Statistical analysis was applied by GraphPad Prism 10 software with an ordinary one-way ANOVA test and Dunnett’s multiple-comparison test. The difference was considered significant for * *p* < 0.05 and ** *p* < 0.01 vs. the negative control group.

## 3. Results and Discussion

**Figure 1:**
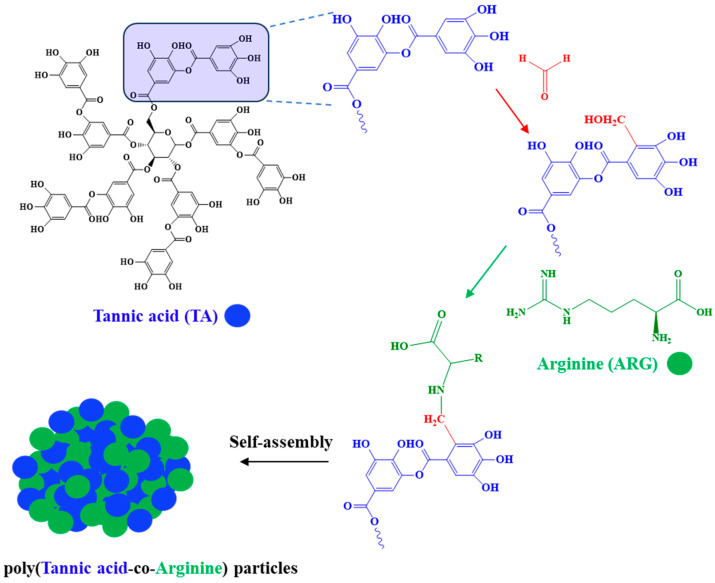
Synthesis of poly(tannic acid-co-arginine) (p(TA-co-ARG)) particles by a Mannich condensation reaction between TA and ARG in the presence of formaldehyde coupling agent.

Tannic acid (TA) is a natural polyphenol that is present in many plants, especially oak and chestnut. It is commonly used in coating, leather, adhesive, and pharmaceutical industries [20], which is related to its excellent antioxidant, antimicrobial, antimutagenic, anticarcinogenic, and homeostatic effects [21,22]. The other component of the prepared particles is an essential amino acid arginine (ARG) containing a cationic functional group in the chemical structure. ARG has substantial biological functions for human health. It is generally found in the active site of proteins and enzymes due to its amine-containing side chain. It is the precursor of nitric oxide synthesis, thus allowing blood vessels to dilate and increasing blood flow rates. Therefore, it is used in the treatment of chest cramps, high blood pressure, and heart diseases. Furthermore, ARG plays a vital role in the detoxification and stimulation of the immune system [23]. Therefore, here poly(tannic acid-co-arginine) particles were prepared to exploit both component biological benefits for biomedical applications. The schematic of p(TA-co-ARG) particles from TA and ARG is presented in Figure 1.

TA is a hydrolyzable ester group affording the polyphenolic structures that consist of ten gallic acid units bounded with a center glucose known as polygalloyl glucose. A formaldehyde van reacted with hydroxyl groups of each gallic acid unit of TA in the first step of the Mannich reaction [24]. Then, in the next step, amine functional groups containing molecules, e.g., ARG, can react with the TA by the formaldehyde-attached side through a Mannich condensation reaction on the methylene bridges as shown in red color in Figure 1 [19]. The ratio of the TA repeating unit and ARG was changed to 1:1, 1:2, and 1:3 to determine reaction mechanisms and biological activities of the obtained p(TA-co-ARG) particles.

**Figure 2:**
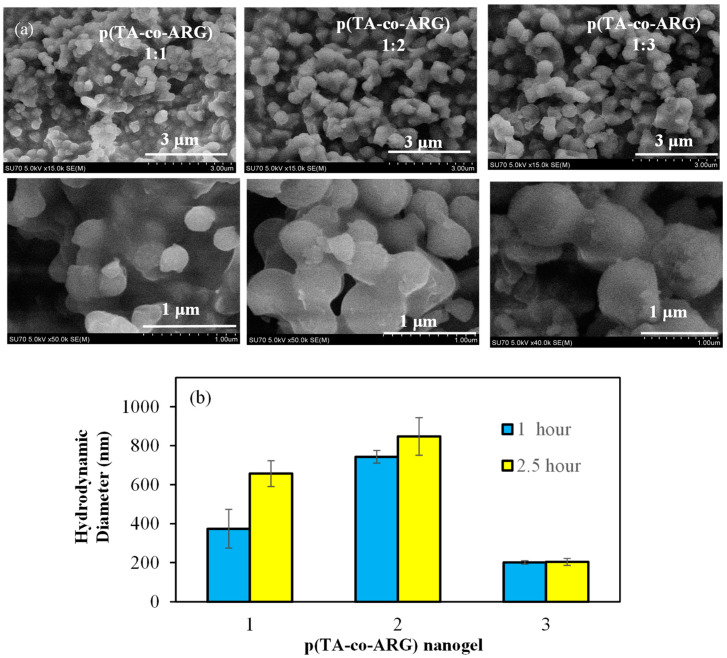
(**a**) SEM images of p(TA-co-ARG) particles prepared at a 1:1, 1:2, and 1:3 mole ratio of TA:ARG and (**b**) size of p(TA-co-ARG) particles prepared at a 1:1, 1:2, and 1:3 mole ratio and prepared at different reaction times (1 h, 2.5 h).

SEM images of p(TA-co-ARG) particles prepared with 1:1, 1:2, and 1:3 mole ratios of TA:ARG are given in Figure 2a. As seen in the SEM images, p(TA-co-ARG) particles are in the few hundred nm size range and have almost spherical shape. The size distribution of the p(TA-co-ARG) particles at a 1:1 mole ratio was approximately 300 nm, but the 1:2 and 1:3 mole ratios of p(TA-co-ARG) particles were found to be nearly 500 and 1000 nm, respectively. It could be said that the size of the p(TA-co-ARG) particles was significantly increased by increasing the ARG ratio in the polymeric structure. One can assume that the size and ARG content of p(TA-co-ARG) particles can be tuned by the amount of ARG as well as other reaction conditions such as mixing rate and FA amount, as well as the time of reaction after the addition of each component.

The size analysis of p(TA-co-ARG) nanoparticles prepared in TA:ARG molar ratios of 1:1, 1:2, and 1:3 was carried out by DLS according to different reaction times. As shown in Figure 2b, the sizes of the p(TA-co-ARG) 1:1, 1:2, and 1:3 nanoparticles in a 2.5 h reaction time were determined to be 657 ± 66, 847 ± 97, and 204 ± 97 nm, respectively. The size of the p(TA-co-ARG) nanoparticles prepared in the TA:ARG molar ratio of 1:1 was 530 + 44 in a reaction time of 1 h. The size of the p(TA-co-ARG) nanoparticles prepared with a TA:ARG molar ratio of 1:3 was not significantly affected by the reaction time. Through the examination of the SEM images and DLS results in Figure 2, one can see that, according to the SEM images, the particles in the water environment are slightly larger. This shows us that the particles are highly water swellable.

**Figure 3:**
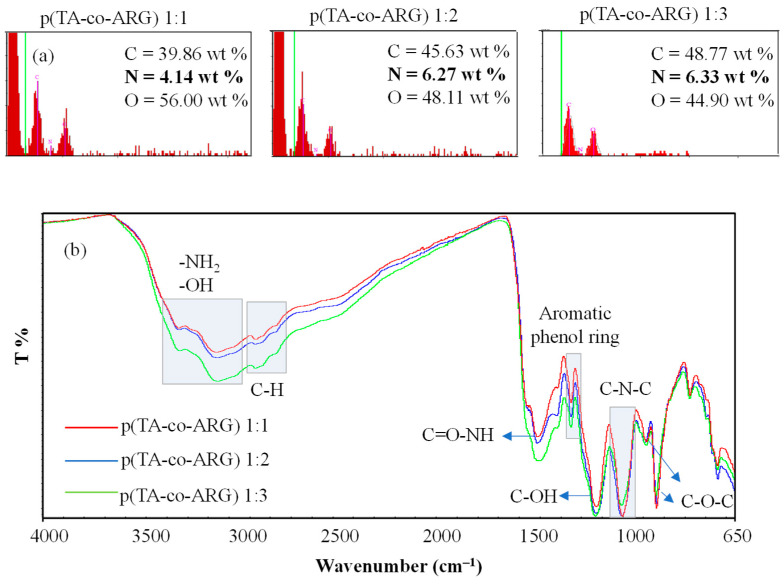
(**a**) SEM–EDX results of p(TA-co-ARG) particles prepared at a 1:1, 1:2, and 1:3 mole ratio and (**b**) their FT-IR spectra.

The chemical composition of the synthesis p(TA-co-ARG) particles was investigated by a scanning electron microscope–energy-dispersive X-ray (SEM–EDX) analysis. The composition ratio of TA:ARG in the p(TA-co-ARG) particle network was changed by changing the mole ratio of TA:ARG, e.g., 1:1, 1:2, and 1:3 during particle synthesis. As shown in Figure 3a, 1:1 mole ratio p(TA-co-ARG) particles were composed of 39.8, 4.1, and 56.0 weight (wt) % of C, N, and O atoms, respectively. The C and N wt % was increased to 45.6 and 6.2 because of the increased amount of ARG in the particle network for p(TA-co-ARG) particles at a 1:2 mole ratio. These results indicate that N% increased from a 1:1 to 1:2 mole ratio of TA:ARG use but was not significantly changed for p(TA-co-ARG) particles synthesis with a 1:3 mole ratio of TA:ARG, indicating that there is a certain limit to the inclusion amount of ARG at the determined reaction conditions. Theoretically, the composition of the N in p(TA-co-ARG) particles at 1:1, 1:2, and 1:3 mole ratios of TA:ARG were calculated as 2.98, 5.46, and 7.01%, respectively. However, N% was determined as 4.14, 6.27, and 6.33% for the same particles, respectively. According to the results, the ARG % of the p(TA-co-ARG) particles was 69.4, 75.7, and 67.7% for the reaction between the 1:1, 1:2, and 1:3 mole ratio of TA:ARG molecules, respectively. Therefore, it could be said that using a 1:2 mole ratio of TA:ARG was enough to increase the ARG in the particle network.

FT-IR spectra of p(TA-co-ARG) particles are given in Figure 3b. The specific peaks of TA in the particles were observed to be centered at about 3375 cm^−1^ for the -OH groups as strong absorption band and in the 3600–3000 cm^−1^ range for the hydroxyl group (O-H) H-bonding as broad strong peaks linked to the aromatic structures. Also, there were stretching frequencies at 2969 and 2875 cm^−1^ belonging to C-H stretching, at 1450 cm^−1^ coming from the phenolic structure, and at 1155 and 1088 cm^−1^ attributable to C-O-C groups [19]. In addition, ARG peaks in the particle structure were seen at 3337 and 3161 cm^−1^ due to N-H stretching vibrations, at 1612 cm^−1^ due to C=O-NH stretching vibrations, at 1333 cm^−1^ due to C-OH bending, and at 1200 cm^−1^ due to C-N-C stretching peaks. It was seen that specific N-H and C-H peaks were gradually increased by increasing the ARG ratio in the particle network, due to the increase amount of ARG incorporation into the particles.

**Figure 4:**
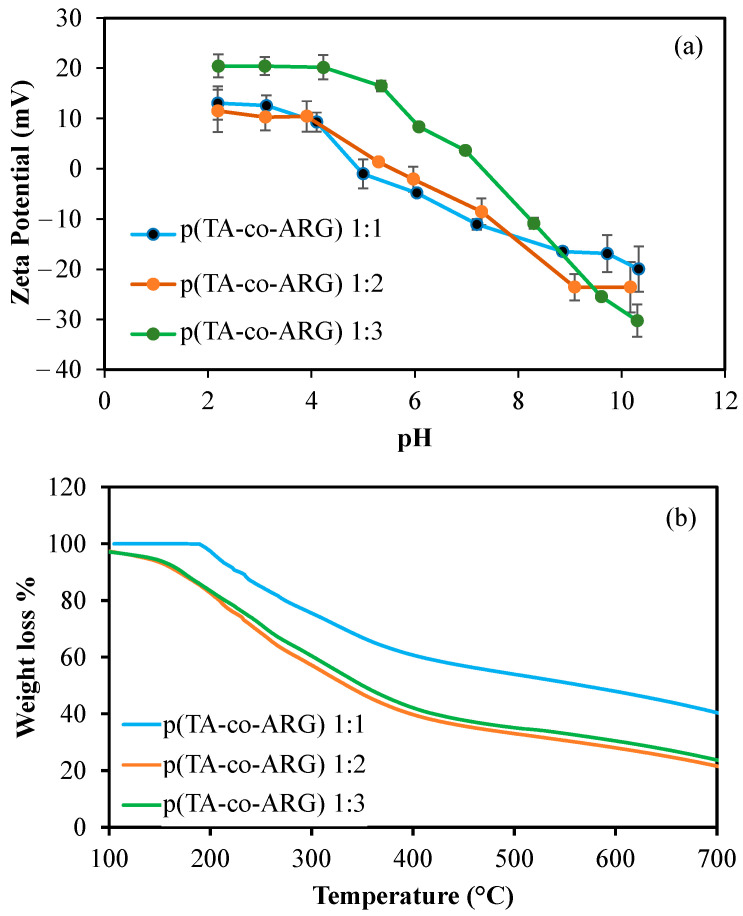
(**a**) Zeta potential values of p(TA-co-ARG) particles prepared at 1:1, 1:2, and 1:3 mole ratios at different pH conditions from pH 2 to pH 10 and their isoelectric points; and (**b**) Thermogravimetric analysis of p(TA-co-ARG) particles prepared at 1:1, 1:2, and 1:3 mole ratios.

Zeta potential values of p(TA-co-ARG) particle suspension were investigated at different pH solutions to estimate the isoelectric point (IEP) of the particles, as illustrated in Figure 4a. The ARG molecule has three pK_a_ values, 2.1, 9.0, and 12.5, which come from the carboxylic acid, amino, and size chain groups of the ARG, and its IEP value is 10.8 [25]. The other component of the particle, TA, has a negative surface charge related to the acidic phenolic hydroxyl groups and it has two pK_a_ values at 4.9 and 8.5 because of different phenolic hydroxyl groups [16]. The zeta potential values of the p(TA-co-ARG) particle at 1:1, 1:2, and 1:3 ratios were measured as −4.8 ± 0.7, −2.1 ± 2.5, and +8.3 ± 0.1 mV, respectively, in an aqueous environment. ARG has a positively charged side chain and enhanced the percentage of positively charged groups in the particle structure when increasing the mole ratio of ARG. Furthermore, the IEP of p(TA-co-ARG) particles 1:1, 1:2, and 1:3 was found as pH 4.98, 5.34, and 7.30, respectively. The increasing IEP values by the increasing ARG ratio are supported by the increasing ARG ratio in the p(TA-co-ARG) particle structure. These results indicated that p(TA-co-ARG) particles show amphoteric structures with both anionic and cationic natures, and the increase in ARG content of the particles brings the IEP close to the physiological pH levels, 7.4. In other words, the p(TA-co-ARG) particles at a 1:3 mole ratio have no net charge at about physiological pHs.

Thermal degradation of the p(TA-co-ARG) particles prepared at 1:1, 1:2, and 1:3 mole ratios is demonstrated in Figure 4a. The p(TA-co-ARG) particles (at 1:1 ratio) revealed two degradation steps at a 195–386 °C range with 37.9% weight loss and at a 400–700 °C range with 59.6% total weight loss. p(TA-co-ARG) particles at a 1:2 ratio also showed two degradation steps, a 152–383 °C range with 65.4% weight loss and a 410–700 °C range with 87% total weight loss. The p(TA-co-ARG) particles at a 1:3 ratio exhibited a similar degradation step with p(TA-co-ARG) particles (1:2) with a 63.1% total weight loss value. These results indicated that the particles at a 1:1 mole ratio are slightly more stable than the 1:2 and 1:3 mole ratio of p(TA-co-ARG) particles, but the thermal degradation profile was not changed for p(TA-co-ARG) particles prepared at 1:2 and 1:3 mole ratios of TA:ARG.

**Figure 5:**
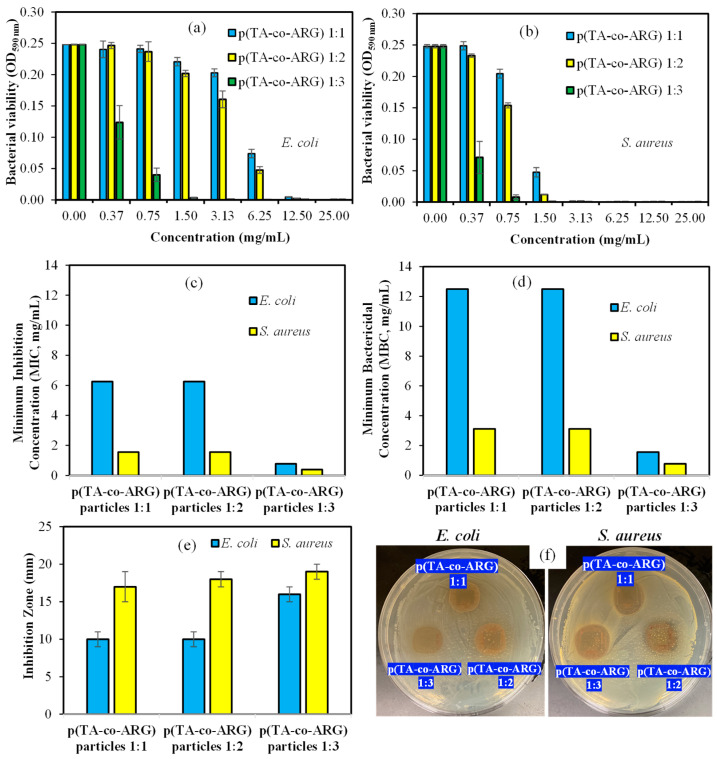
Bacterial viability of p(TA-co-ARG) particles at 1:1, 1:2, and 1:3 mole ratios against (**a**) *Escherichia coli* (ATCC 8739) as Gram-negative bacteria and (**b**) *Staphylococcus aureus* (ATCC 6538) as Gram-positive bacteria, as well as their (**c**) minimum inhibition concentration (MIC), (**d**) minimum bactericidal concentration (MBC), (**e**) inhibition zone values for 20 μL of 50 mg/mL p(TA-co-ARG) particles suspension, and (**f**) corresponding particles’ pictures in Petri dishes for *E. coli* and *S. aureus* microorganism.

It is well known that TA is an antimicrobial compound against a wide range of microorganisms [21]. Similarly, ARG inhibits the bacterial growth of bacteria due to its cationic nature and shows great potential in wound-healing applications [26]. The antibacterial activity of p(TA-co-ARG) particles prepared at 1:1, 1:2, and 1:3 mole ratios were investigated by microtiter dilution and disc diffusion assays. A bacterial viability test of p(TA-co-ARG) particles at 1:1, 1:2, and 1:3 mole ratios with varying concentration ranges against Gram-negative *Escherichia coli* (ATCC 8739) and Gram-positive *Staphylococcus aureus* (ATCC 6538) at 24 h incubation time was performed, and the corresponding results are shown in Figure 5a and Figure 5b, respectively. Also, the MIC and MBC values of the particles against *E. coli* and *S. aureus* are given in Figure 5c,d.

As can be seen in the Figures, all forms of p(TA-co-ARG) particles afforded gradual bacterial inhibition proportional to the increasing particle concentration. According to the results, 6.25 mg/mL MIC and 12.5 mg/mL MBC values of p(TA-co-ARG) particles prepared at 1:1 and 1:2 mole ratios resulted in almost the same effect against *E. coli*, but p(TA-co-ARG) particles at a 1:3 ratio exhibited a more potent antibacterial effect with 0.78 mg/mL MIC and 1.56 mg/mL MBC values against *E. coli*. P(TA-co-ARG) particles showing more potent antibacterial effects on *S. aureus* (Gram-positive bacteria) with 1.56 mg/mL MIC and 3.12 mg/mL MBC values for 1:1 and 1:2 p(TA-co-ARG) particles and 0.39 mg/mL of MIC and 0.78 mg/mL of MBC values for 1:3 p(TA-co-ARG) particles. In the other antibacterial test, the inhibition zone of 1 mg p(TA-co-ARG) particles at 1:1, 1:2, and 1:3 ratios were found as 10 ± 1, 10 ± 1, and 16 ± 1 mm against *E. coli* and 17 ± 2, 18 ± 1, and 19 ± 1 mm against *S. aureus*, as shown Figure 5e, and the corresponding photographs are given in Figure 5f. Sahiner et al. reported that TA had MIC values of 10 and 1 mg/mL against *E. coli* and *S. aureus*, respectively [21]. No strong antibacterial activity was reported for ARG amino acid, e.g., 100 and 25 mg/mL MIC values against *E. coli* and *S. aureus*, respectively [27,28]. However, the polypeptide or polymeric forms of ARG-derived structure exhibited impressive antibacterial potency against Gram-negative and Gram-positive bacteria [29]. Similarly, 1 mg of TA revealed 12 mm and 18 mm inhibition zones against *E. coli* and *S. aureus*, respectively, whereas 10 mg of L-ARG had a low bacterial inhibition ability, e.g., only against Gram-positive bacteria, *S. aureus*, with a 10 mm inhibition zone [28]. These results supported that the antibacterial effectiveness of TA and ARG can be significantly improved by preparing their corresponding particle forms. Therefore, using this phenolic compound TA and the molecule amino acid ARG to make particles, a synergistic antibacterial effect against both types of microorganisms can be attainable, as demonstrated in this investigation. Moreover, the antibacterial potency of p(TA-co-ARG) particles is increased by the increasing ARG content in the particle structure. These particles had a greater inhibition effect on Gram-positive bacteria than Gram-negative bacteria.

**Figure 6:**
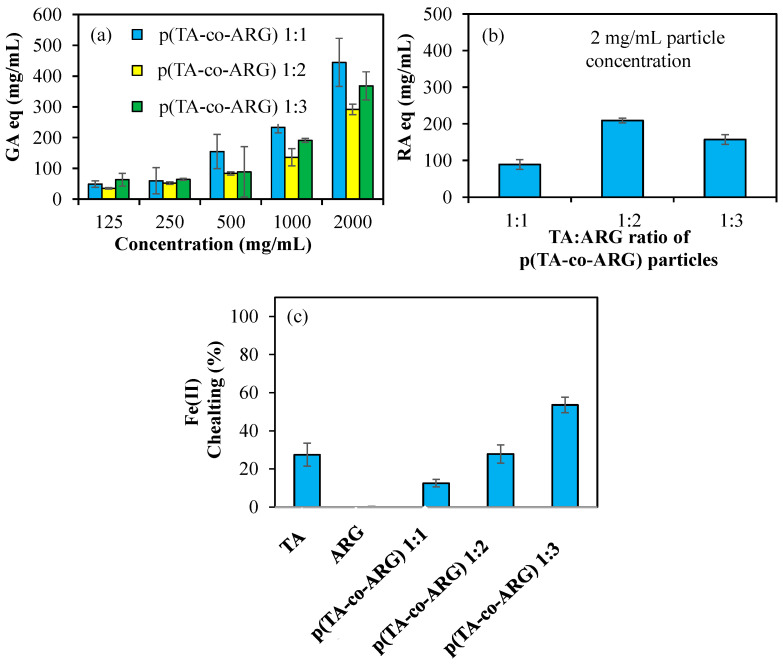
(**a**) Total phenol content of p(TA-co-ARG) particles prepared at 1:1, 1:2, and 1:3 mole ratios at different concentrations, (**b**) total flavonoid content of (TA-co-ARG) particles prepared at 1:1, 1:2, and 1:3 mole ratios at a concentration of 2 mg/mL, and (**c**) Fe(II) chelating capability (%) of TA, ARG, and p(TA-co-ARG) particles prepared at 1:1, 1:2, and 1:3 mole ratios at 2 mg/mL concentration.

Figure 6a shows the antioxidant properties of p(TA-co-ARG) nanoparticles prepared at 1:1, 1:2, and 1:3 ratios tested at varying concentrations of 125–2000 μg/mL according to the FC test. P(TA-co-ARG) particles at a 1:1 ratio have the highest antioxidant properties. For a concentration of 1000 µg/mL, it has 234 ± 18, 136 ± 28, and 190 ± 7 mg/mL GA equivalent values for 1:1, 1:2, and 1:3, respectively. In addition, according to the FC test, the antioxidant properties increase depending on the concentration.

Figure 6b shows the antioxidant properties of p(TA-co-ARG) nanoparticles at concentrations of 2 mg/mL according to the TFC test. P(TA-co-ARG) at 1:2 particles have 210 ± 6 RA equivalent, which has the highest antioxidant properties. Figure 6c shows the 1:1, 1:2, and 1:3 Fe(II) chelating capacities of TA, ARG, and p(TA-co-ARG) nanoparticles at concentrations of 250 µg/mL. Figure 6c shows the Fe(II) chelating capacities of TA, ARG, and p(TA-co-ARG) nanoparticles at 250 µg/mL concentrations for 1:1, 1:2, and 1:3 ratios. TA had a chelating capacity of 27 ± 6% at 250 mg/mL, while ARG did not chelate Fe(II). p(TA-co-ARG) at a 1:3 ratio chelated Fe(II) at a ratio of 54 ± 4%. p(TA-co-ARG) particles have a greater chelating capacity than TA molecules; this can be explained by the fact that TA molecules are in the form of particles that are nanonetwork in 3D, allowing the functional groups to interact at any angle with Fe(II) ions.

**Figure 7:**
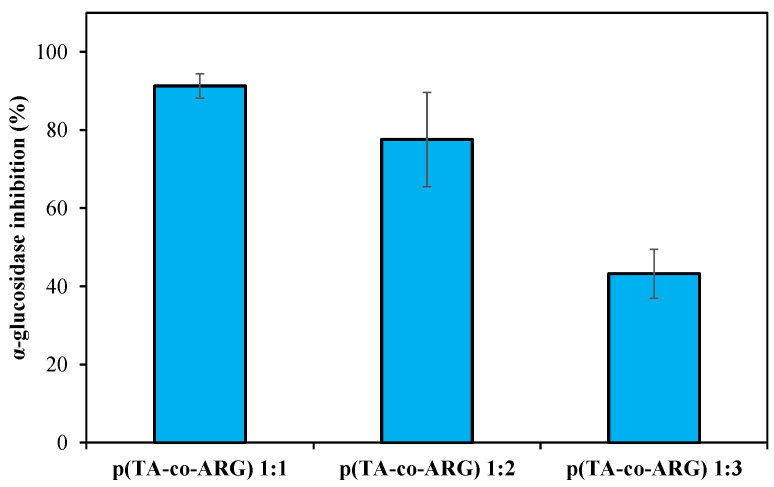
The α-glucosidase inhibition of p(TA-co-ARG) nanogels at 2 mg/mL concentration.

It is known that polyphenols inhibit the α-glucosidase enzyme [30,31]. The ability of polyphenols to suppress the activity of digestive enzymes, thus postponing the hydrolysis of starch, makes them a potential antidiabetic functional material [30]. Figure 7 demonstrates the inhibition graph of α-glucosidase enzyme with 2 mg/mL concentrations of p(TA-co-ARG) nanoparticles at 1:1, 1:2, and 1:3 ratios. The highest inhibition value was 91.3 ± 3.1% inhibition by p(TA-co-ARG) particles prepared at a 1:1 mole ratio. The p(TA-co-ARG) particles (1:2) inhibited 77.6 ± 12.0% α-glucosidase enzyme. As the ARG ratio in particle synthesis increases, enzyme inhibition decreases as expected.

**Figure 8:**
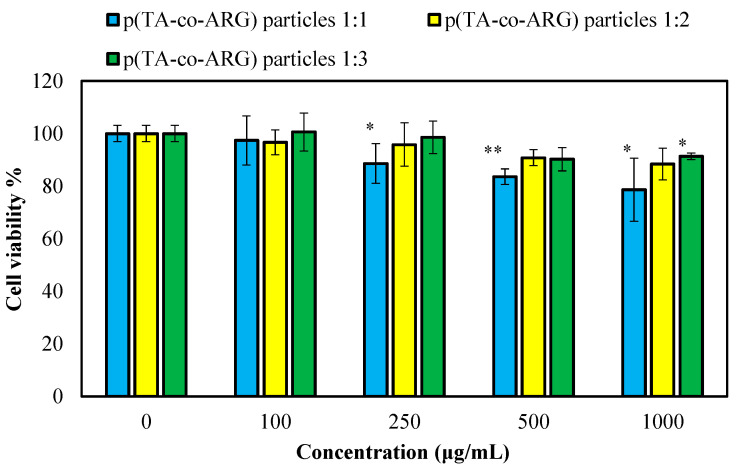
Cytotoxicity of p(TA-co-ARG) particles prepared at 1:1, 1:2, and 1:3 mole ratios on L929 fibroblast cells for a 24 h incubation time. The *p*-values as * *p* < 0.05 and ** *p* < 0.005 vs. control were given as statistically significant.

The toxicity of the prepared p(TA-co-ARG) particles was determined on L929 healthy fibroblast cells, and the results are presented in Figure 8. All forms of particles were found to be non-toxic on the fibroblasts at a 100 µg/mL concentration, with more than 93% cell viability. As expected, at all concentrations between 250 and 1000 particle concentration, the p(TA-co-ARG) particles at 1:2 and 1:3 mole ratios showed >90% cell viability.

The cell viability of the p(TA-co-ARG) particles prepared at 1:1, 1:2, and 1:3 ratios was found to be 79 ± 12, 88 ± 6, and 91 ± 1%, respectively, for 1000 μg/mL concentration. As reported by our previous studies, TA has a cytotoxic effect on fibroblast cells, even at a 10 μg/mL concentration [21]. As anticipated, ARG is not toxic on the fibroblast up to a 100 μg/mL concentration, and this information is given in Supplementary Figure S1. It could be said that the toxicity of the p(TA-co-ARG) particles was slightly decreased with increasing ARG content. All of these p(TA-co-ARG) particles have great potential for biomedical use due to their lower toxicity even at high concentrations.

## 4. Conclusions

The natural polyphenol TA and the essential amino acid ARG were used to prepare p(TA-co-ARG) particles by means of a Mannich condensation reaction. The mole ratio of TA:ARG was changed from 1:1 to 1:3 to attain p(TA-co-ARG) particles with variable size, which was significantly increased from 300 nm to 1000 nm and variable in ARG content. A DLS analysis corroborated that p(TA-co-ARG) particles are highly swellable in an aqueous solution. The p(TA-co-ARG) particles with an increased amount of ARG content resulted in higher antibacterial properties with slightly lower antioxidant properties as well as improved biocompatibility. Additionally, the IEP of the particles increased to a pH value of 7.3 for p(TA-co-ARG) particles at a 1:3 ratio of TA:ARG because of the high content of cationic ARG molecules, making this particle very close to physiological pHs. P(TA-co-ARG) particles show antioxidant properties according to three different antioxidant tests, which are GA, RA, and Fe(II) chelating ability. Also, p(TA-co-ARG) particles have α-glucosidase inhibition properties, which are very important in diabetic patients; e.g., these particles can be used for treatment of type II diabetes mellitus. Moreover, the higher antibacterial activity capabilities of p(TA-co-ARG) particles against both Gram-positive and Gram-negative bacteria make them suitable for antipathogenic application or prevention of infection caused by various microorganisms. Moreover, all types of p(TA-co-ARG) particles are highly biocompatible, making them great candidates for in vivo drug delivery materials.

## Data Availability

All data generated in this research are contained within this research.

## Supplementary Materials

The following supporting information can be downloaded at: https://www.mdpi.com/article/10.3390/biomedicines13010209/s1, Figure S1: Cytotoxicity of L-arginine on L929 fibroblast cells for a 24 h incubation time.
