# Supplementary material for: Nanoparticles for Biomedical Use Derived from Natural Biomolecules: Tannic Acid and Arginine
Source: Biomedicines. 2025 Jan 16;13(1):209. doi: 10.3390/biomedicines13010209 (PMC11762816; doi:10.3390/biomedicines13010209)
Supplement: Supplementary file 1 [file biomedicines-13-00209-s001.zip › biomedicines-3427146-supplementary.pdf]

Nanoparticles for Biomedical Use Derived from Natural Biomolecules: Tannic Acid and Arginine

Mehtap Sahiner [1], Selin S. Suner [2] and Nurettin Sahiner [2,3,*]

[1] Department of Bioengineering, Faculty of Engineering, Canakkale Onsekiz Mart University, Terzioglu Campus, Canakkale 17199, Turkey; sahinerm78@gmail.com
[2] Department of Chemistry, Faculty of Sciences, Canakkale Onsekiz Mart University, Terzioglu Campus, Canakkale 17100, Turkey; sagbasselin@gmail.com
[3] Department of Bioengineering, U.A. Whitaker College of Engineering, Florida Gulf Coast University, Fort Myers, FL 33965, USA
[*] Correspondence: sahiner71@gmail.com or nsahiner@fgcu.edu

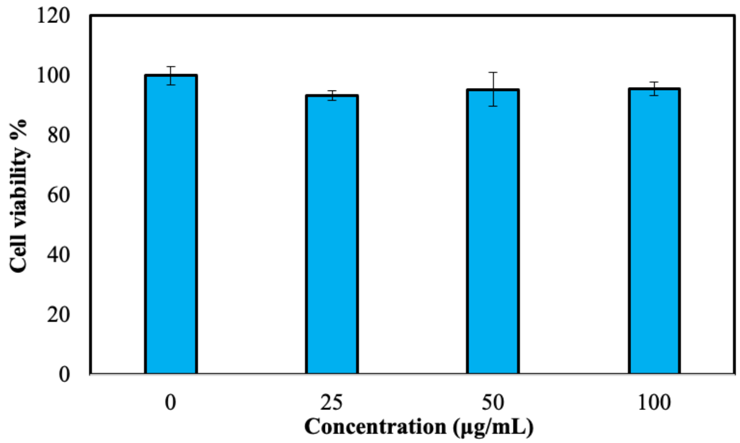

Figure S1. Cytotoxicity of L-Arginine on L929 fibroblast cells for 24 h incubation time.
